# Ocean warming and Marine Heatwaves unequally impact juvenile introduced and native oysters with implications for their coexistence and future distribution

**DOI:** 10.1038/s41598-024-71534-9

**Published:** 2024-09-05

**Authors:** Nate Howarth, Elliot Scanes, Maria Byrne, Pauline M. Ross

**Affiliations:** 1 School of Life and Environmental Sciences, The University of Sydney, Camperdown, Sydney, NSW 2006 Australia; 2 Climate Change Cluster, University of Technology, Ultimo, Sydney, NSW 2007 Australia

**Keywords:** Climate-change ecology, Climate-change ecology

## Abstract

Climate change is causing ocean warming (OW) and increasing the frequency, intensity, and duration of extreme weather events, including Marine Heat Waves (MHWs). Both OW and MHWs pose a significant threat to marine ecosystems and marine organisms, including oysters, oyster reefs and farmed oysters. We investigated the survival and growth of juveniles of two commercial species of oyster, the Sydney rock oyster, *Saccostrea glomerata*, and the Pacific oyster, *Crassostrea gigas*, to elevated seawater temperatures reflecting a moderate and an extreme MHW in context with recent MHWs and beyond. The survival and size of Pacific oysters to moderate MHWs (22–32 °C; 14 days) was greater than that for Sydney rock oysters (24–32 °C; 15 days). While survival and growth of both species was significantly impacted by extreme MHWs (29–38 °C; 5–6 days), Sydney rock oysters were found to survive greater temperatures compared to the Pacific oyster. Overall, this study found that Pacific oyster juveniles were more tolerant of a moderate MHW, while Sydney rock oyster juveniles were more resilient to extreme MHWs. These differences in thermal tolerance may have consequences for aquaculture and coexistence of both species in their intertidal and latitudinal distributions along the south-eastern Australian coastline.

## Introduction

Anthropogenic driven climate change is causing ocean warming (OW). Worse case climate models (Shared Socioeconomic Pathways, SSP 5–8.5) project that by the end of this century chronic OW will occur, and the mean Sea Surface Temperatures (SST) will rise to nearly 3 °C (SSP5-8.5 range 2.01–4.07 °C)^1^. Along with OW there will be an increase in the frequency, intensity, and duration of extreme weather and climatic events in the oceans such as Marine Heat Waves (MHWs). While the impacts of atmospheric heat waves in terrestrial ecosystems are well studied^2^, there remains much to be uncovered about the impacts of OW and atmospheric and MHWs on marine ecosystems and organisms^1,3–6^. MHWs are defined as a prolonged periods where SST are anomalously high, equal to and above the 90th percentile, and persist for five days or longer^5^.

Since last century, there has been a greater than 50% increase in MHWs^7,8^. Notable examples of MHWs events include the MHW in 2003 in the Northwestern Mediterranean region where SST reached 1–3 °C above mean and maximum values ever recorded and caused extensive mortality of 25 marine benthic organisms (mainly gorgonians and sponges)^9^. In 2010–2011 a MHW event along the coastline of Western Australia where SST reached 3 °C above the monthly mean^10^ caused extensive mortality of approximately 36% of the seagrass meadow in Shark Bay^11,12^ which is yet to fully recover^13^. In 2013–2015, a MHW nicknamed ‘The Blob’ off the coast of the north-eastern Pacific caused mass strandings and deaths of marine mammals, fish, krill, and seabirds and geographical range shifts of marine organisms^14^. This MHW event also caused reduced spawning and/or failure to spawn in several taxa with implications for recruitment and reproduction^15^. Australia’s Great Barrier Reef has had five major MHW driven mass coral bleaching and mortality events since 2015 with the 2015–2016 MHW event lasting 318 days^16,17^. The increasing frequency of MHWs does not give corals the recovery time needed, placing coral reefs in a perilous state^17,18^. It is predicted that MHWs will become even more frequent and extreme^19,20^ with rapid and catastrophic consequences for marine ecosystems and organisms^16,20–24^. The impacts of MHWs have also been severe for farmed animals raising economic and food security concerns^25,26^ as seen in mortality of abalone (*Haliotis roei*), scallops (*Amusium balloti*), prawns (*Penaeus latisulcatus; P. esculentus*) and oysters^27–30^.

Oysters play two vital roles in coastal ecosystems globally. They form the basis of aquaculture providing a source of protein and income for communities world-wide^31^, and oyster reefs. As ecosystem engineers, oysters create biogenic habitat^32^, provide ecosystem services such as nutrient and nitrogen cycling^33,34^, and shelter and spawning substrate for a variety of species^35^. They are also a preyed on by many species^36^ including fish^37^, whelks and crabs^38^. However, 85% of oyster reefs have been lost globally due to overfishing, introduced pests and poor water quality^39^. In south-eastern Australia 90% of oyster reefs have been lost with the quality of remaining reefs classified as poor and considered critically endangered^40^.

While OW and MHWs threaten the aquacultural and ecosystem services that oysters provide, impacts may not be equal among species^41^ and interactions among species are likely to be altered^42^, which may lead to shifts in distributions^4,43–46^. There remains, however, a lack of knowledge on oyster species responses to OW and MHWs. For south-eastern Australian this is concerning because this region is a climate change “hot spot”^47^ and is also the location where the native Sydney rock (*Saccostrea glomerata*) and the introduced Pacific oyster (*Crassostrea gigas*) co-occur and are commercially important. The capacity to better predict how introduced and native species interact and whether this will be changed by OW and MHW is critical to create effective adaptive strategies^42,48^.

It is reasonable to predict that with warmer seawater temperatures, Pacific oysters may outcompete the native Sydney rock oyster. Pacific oysters are known for their survival across a wide range of temperatures from < 3 to 35 °C^49–52^ and in the northern hemisphere have outperformed competitors^53–55^. Pacific oysters although native to the Northwest Pacific, were introduced from Japan across the world to become a leading global shellfish aquacultural species^56,57^. These oysters have invaded many regions in Europe where they outcompete native species such as native blue mussel, *Mytilus edulis*^53–55^, cause shifts in benthic suspension-feeder populations^54^, and impact food webs and predators such as birds^54^. The invasive distribution is significant, for example, in the Netherlands, approximately 90% of dykes in the Oosterschelde estuary have been colonised by *C. gigas*^58^.

**Fig. 1:**
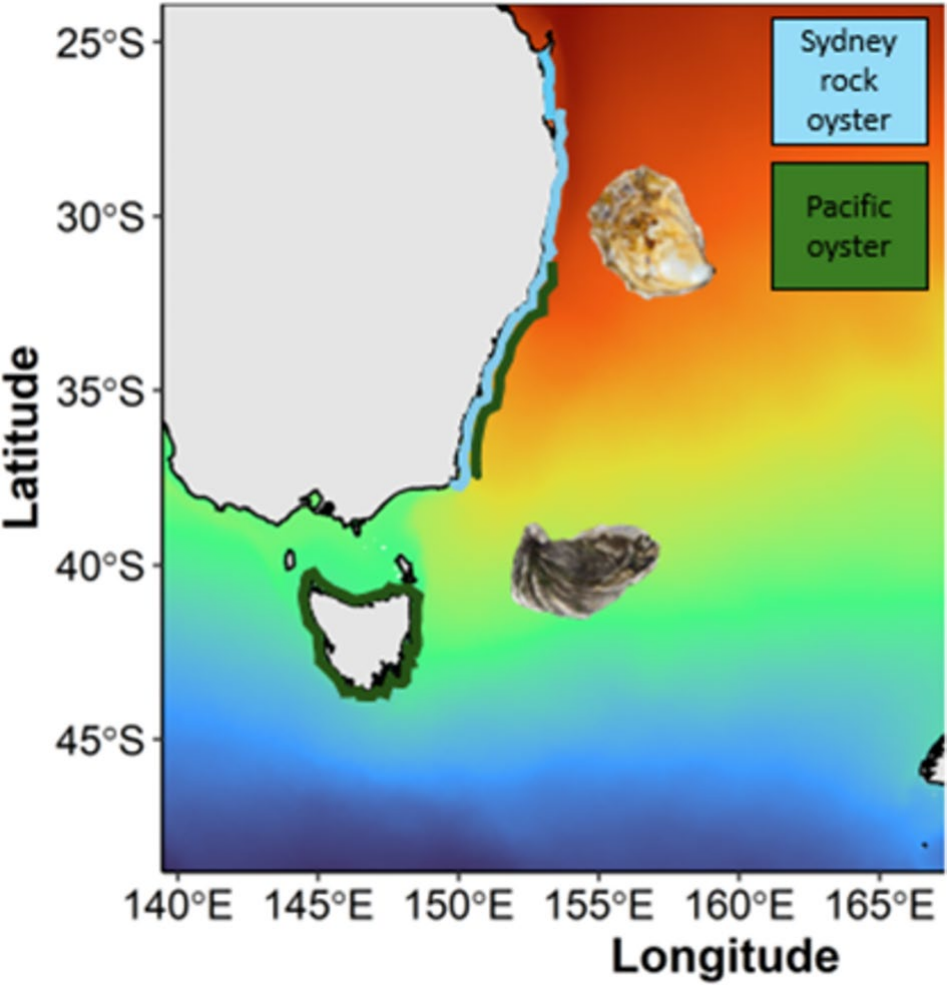
Distribution of the Pacific oyster (*Crassostrea gigas;* purple) and Sydney rock oyster (*Saccostrea glomerata;* blue) on the south-eastern coast of Australia^89,94^. Mean sea surface temperature (SST) is shown by shading.

In Australia, the native Sydney rock oyster is an important aquacultural species and the dominant oyster species in intertidal and subtidal ecosystems^46,59,60^ with a distributional range from the temperate border of Victoria to the tropical climatic range of Queensland (Fig. 1). Historically, Sydney rock oysters were sustainably collected and consumed by First Nations Australians^61,62^. Early European settlers, however, significantly reduced oyster populations through large scale harvesting for food and building construction. Also, since the 1960s Sydney rock oysters have also been under increasing pressures from pollution, poor water quality, sedimentation and novel diseases which have resulted in further declining populations and aquaculture production^63–67^. Today, climate change adds to these pressures on the Sydney rock oyster with the additional threat of OW and MHWs which may lead to potentially altered interactions with the introduced Pacific oyster. Studies have found that larvae of Pacific oysters outperform Sydney rock oysters and can withstand elevated temperatures of 30 °C^41,68,69^. The extent to which oysters can withstand OW and MHWs and any variation between oyster species remains unknown.

Australia’s southeastern estuaries are rapidly warming at rates above those expected for oceanic waters^69^. In these emerging conditions it is not known if the Sydney rock oyster can maintain its status as the dominant oyster species, or be displaced by the invasive challenger, the Pacific oyster. Understanding responses of these two oyster species to OW and MHW has never been more pressing and is needed to inform conservation and reef restoration efforts of Sydney rock oysters. For oyster reef restoration projects to be successful long term, will require oysters which can tolerate OW and more frequent and intense MHWs^45^.

We investigated the survival and growth of juvenile oysters as they are a critical transitionary life stage and are a mortality bottleneck for benthic marine invertebrates and oyster aquaculture^69^. The success of newly settled recruits determines whether a population of oyster can or cannot persist^70,71^. We predicted that given the more northerly (warm affinity) distribution of the Sydney rock oyster, it will have greater growth and survival in response to OW and MHWs compared to the Pacific oyster (Fig. 1).

## Results

### Sydney rock oyster

**Fig. 2:**
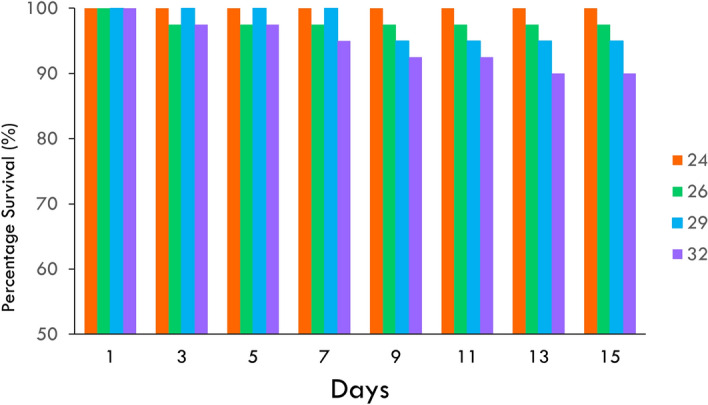
Percentage survival of Sydney rock oyster juvenile, *Saccostrea glomerata*, at four temperatures (24–32 °C) for 15 days (9/8/2023–23/08/2023).

**Fig. 3:**
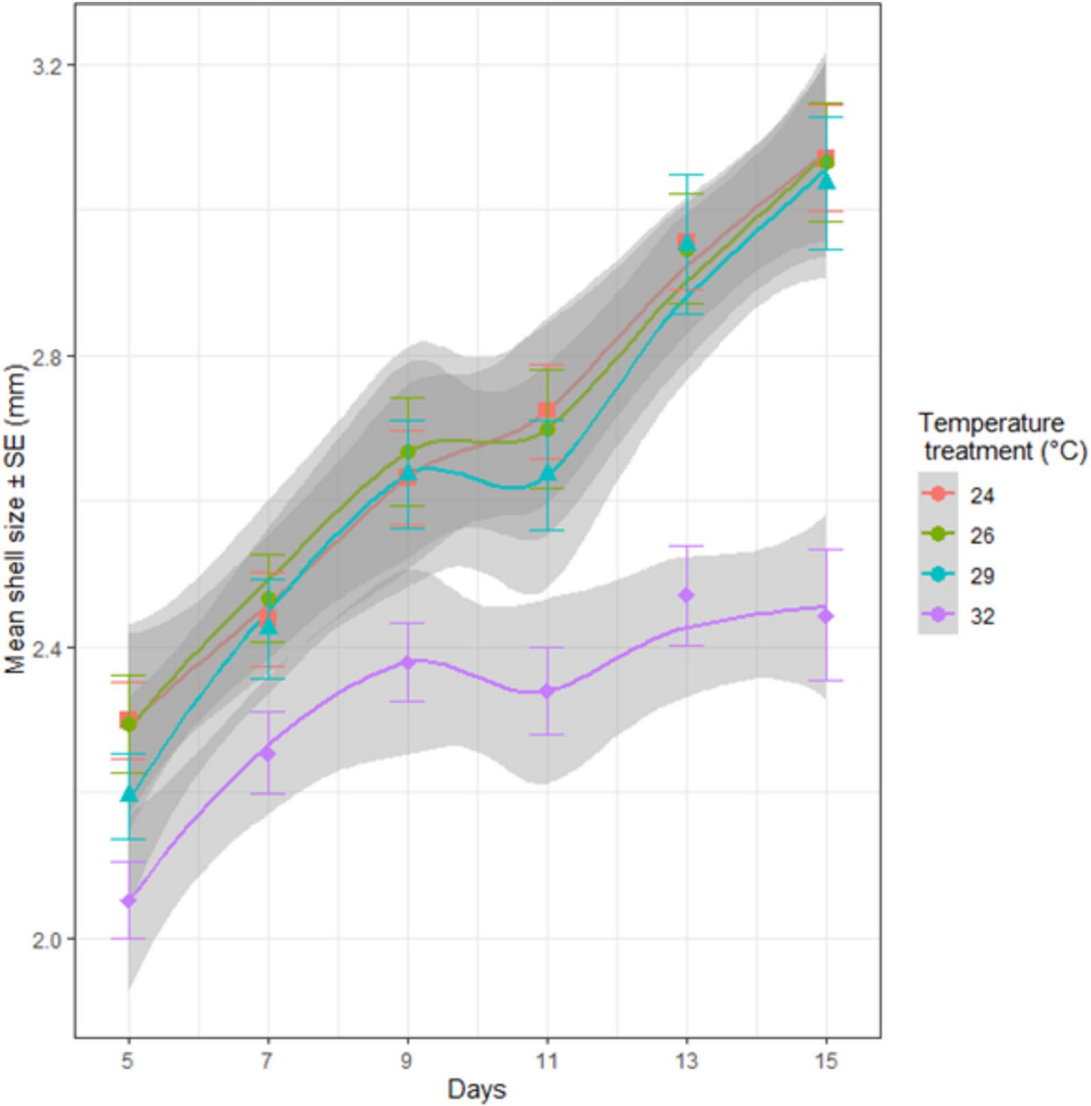
Shell size (mm) (mean ± SE) of juvenile Sydney rock oysters, *Saccostrea glomerata*, at four temperatures from 24 to 32 °C across a 15 day experiment.

**Fig. 4:**
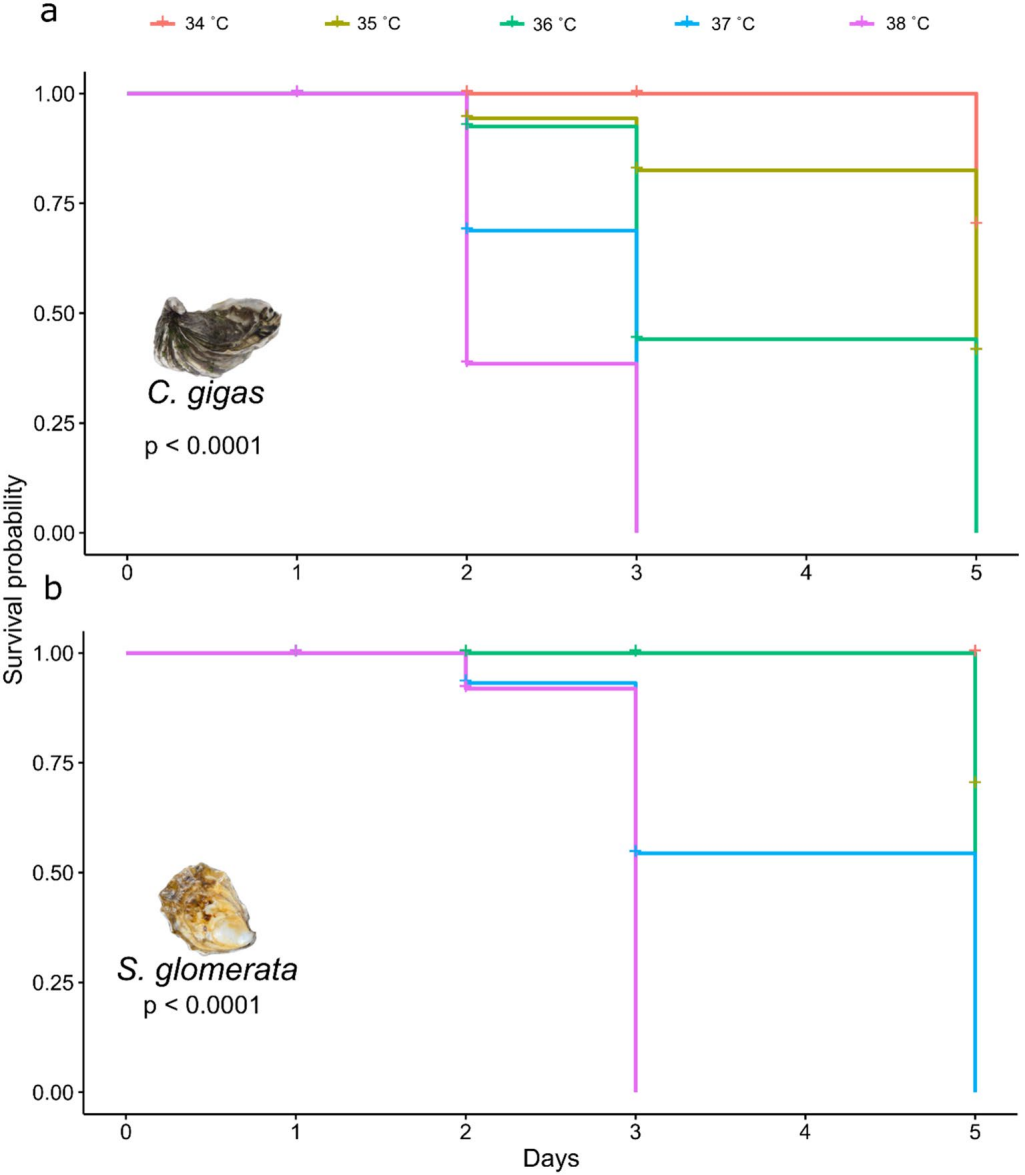
Survival probability modelled using Cox proportional survival in the upper temperature treatments of 34, 35, 36, 37, and 38 °C across 5-days for juvenile Pacific oysters (**a**) and Sydney rock oysters (**b**). Only temperatures with mortality were included.

**Fig. 5:**
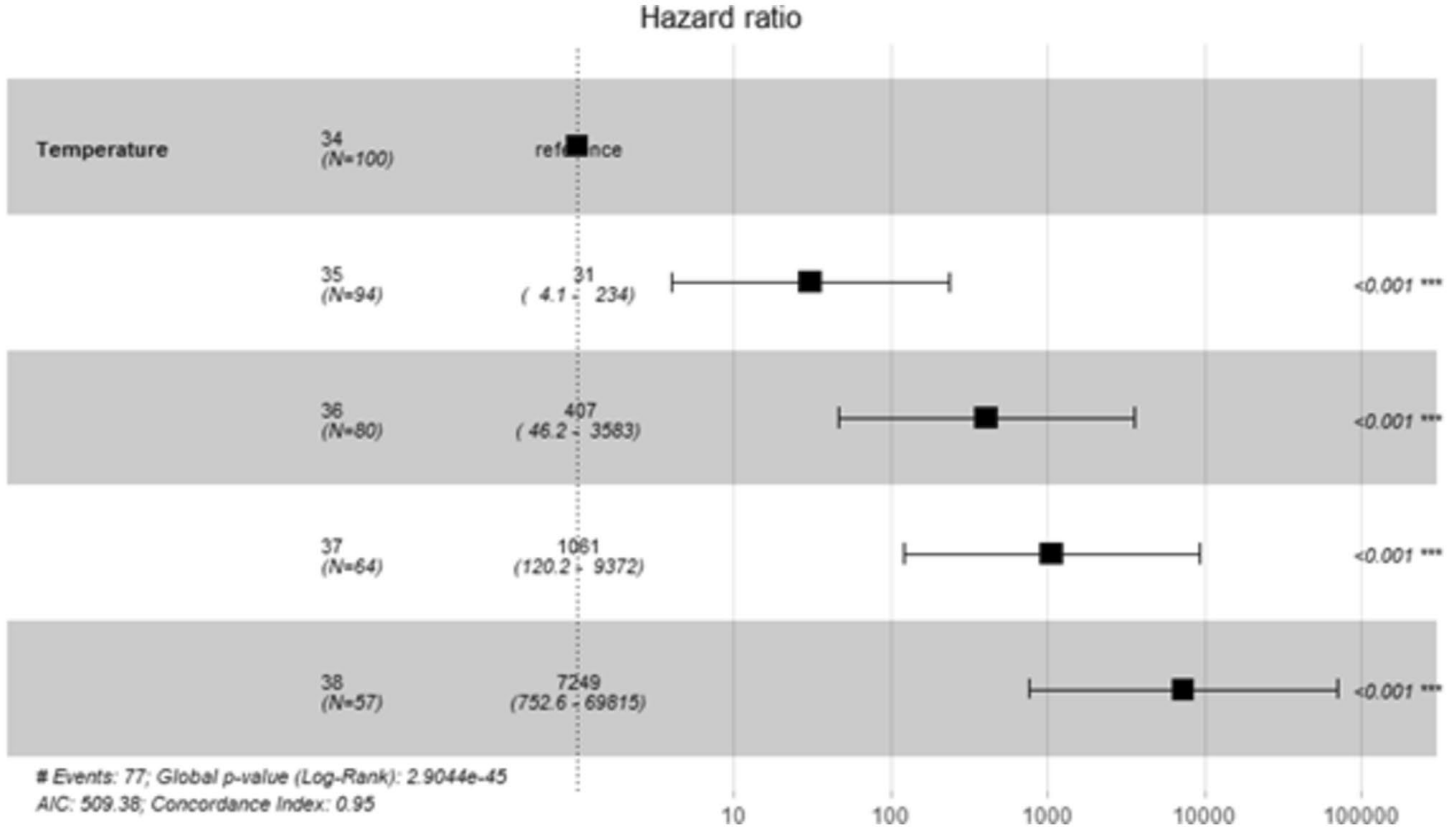
Forrest plot of hazard ± 95% Confidence intervals, measured as mortality of juvenile *S. glomerata* at 34–38 °C across the 6-day experiment (7/09/2023–12/09/2023). *P* values indicate significant differences from the 34 °C treatment.

In the moderate MHW, survival of Sydney rock oyster juveniles was 100% at 24 °C and 90% at 32 °C at day 15 (Fig. 2, Table 1). Although there was a trend for greater mortality at 32 °C, this was not significant even for the prolonged period of 15 days (*p* > 0.05, Fig. 2). While survival was not affected by temperature, juveniles were significantly smaller at the highest temperature of 32° from day five onwards, but similar in size at the three lower temperatures of 24°, 26°, and 29° (Fig. 3, Tables 1, 2). In the extreme MHW there was no survival of Sydney rock oyster juveniles at 38 °C by day three and similarly no survival of juveniles at 37 °C and 36 °C by day five (Fig. 4). While *S. glomerata* juveniles did survive at 34 °C and 35 °C, survival at 35 °C was significantly less than at 34 °C at day six (Fig. 5). The risk of Sydney rock oyster juvenile mortality, as analysed using Cox’s Proportional Hazard model, was significantly heightened at 35–38 °C compared to 34 °C, with juveniles at the highest temperature of 38 °C being over 7249 times more likely to suffer mortality compared to juveniles at 34 °C (Fig. 5, Table 3). However, these hazard ratios are largely not indicative because we saw no mortality in Sydney rock oysters at the temperature of 34 °C. The LT_50_ value for Sydney rock oysters was 36.9 ± 5.5 at day 3, and 35.05 ± 20 at day 5. In contrast to survival the mean size of Sydney rock oyster juveniles was not significantly different at any temperature from 29 to 38 °C. This was because many juveniles did not survive long enough to be measured at the highest temperatures.

**Table 1:** Summary of experiments on juvenile Sydney and Pacific oysters from August–October 2023.

MHW and species	Temperature range °C	Initial size (μm)	Date	Time period, replication and number of oysters
Moderate MHW				
Sydney rock oyster, *S. glomerata*	24–32 °C	1200	9/8/2023–23/08/2023	15 Days n = 8 (total 160)
Pacific oyster, *C. gigas*	22–32 °C	1200	26/09/2023–09/10/2023	14 Days n = 8 (total 200)
Extreme MHW				
Sydney rock oyster, *S. glomerata*	29–38 °C	2000	7/09/2023–12/09/2023	6 Days n = 4 (total 200)
Pacific oyster, *C. gigas*	29–38 °C	1200	10/10/2023–14/10/2023	5 Days n = 4 (total 200)

**Table 2:** Shell size of juvenile Sydney rock oysters, *Saccostrea glomerata*, in a 15 day moderate MHW.

Day	5				11			15		
	Df	MS	F	*P*	MS	F	P	MS	F	P
Temperature	3	0.53	4.03	0.008*	1.18	6.18	0.001***	3.47	13.12	1.21e−07***
Residual	154	0.13			0.19			0.26		
Post hoc	32 > 24 = 26 = 29				32 > 24 = others			32 > 24 = others		

This was a single factor analysis of variance with temperature (24–32 °C) as a fixed factor. Significance labelled as *P* < 0.001 ‘***’, *P* < 0.01 **, *P* < 0.05*. Non significant differences are labelled as ‘ns’.

**Table 3:** Cox’s proportional hazard model measuring hazard as mortality of juvenile Sydney rock oysters, *S. glomerata*, in an extreme MHW using 34 °C as the basis for comparisons with other temperatures.

Temperature °C	Coefficient	Hazard ratio	Std. Err. coefficient	z	P (z)	
35 °C	1.670e + 01	1.793e + 07	4.642e−01	35.98	< 2e−16	***
36 °C	1.866e + 01	1.276e + 08	2.852e−01	65.45	< 2e−16	***
37 °C	1.962e + 01	3.333e + 08	2.771e−01	70.82	< 2e−16	***
38 °C	2.156e + 01	2.308e + 09	3.806e−01	56.65	< 2e−16	***

Significance labelled as *P* < 0.001 ‘***’, *P* < 0.01 **, *P* < 0.05*. Non significant differences are labelled as ‘ns’.

### Pacific oyster

**Fig. 6:**
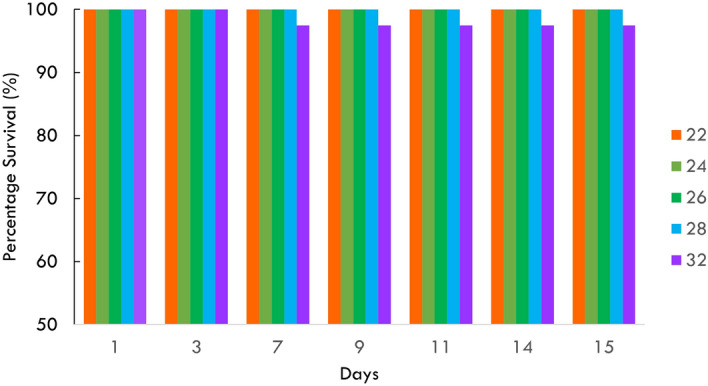
Percentage survival of juvenile Pacific oyster, *Crassostrea gigas* at five treatments for at temperatures for 14 days (26/09/2023–09/10/2023).

**Fig. 7:**
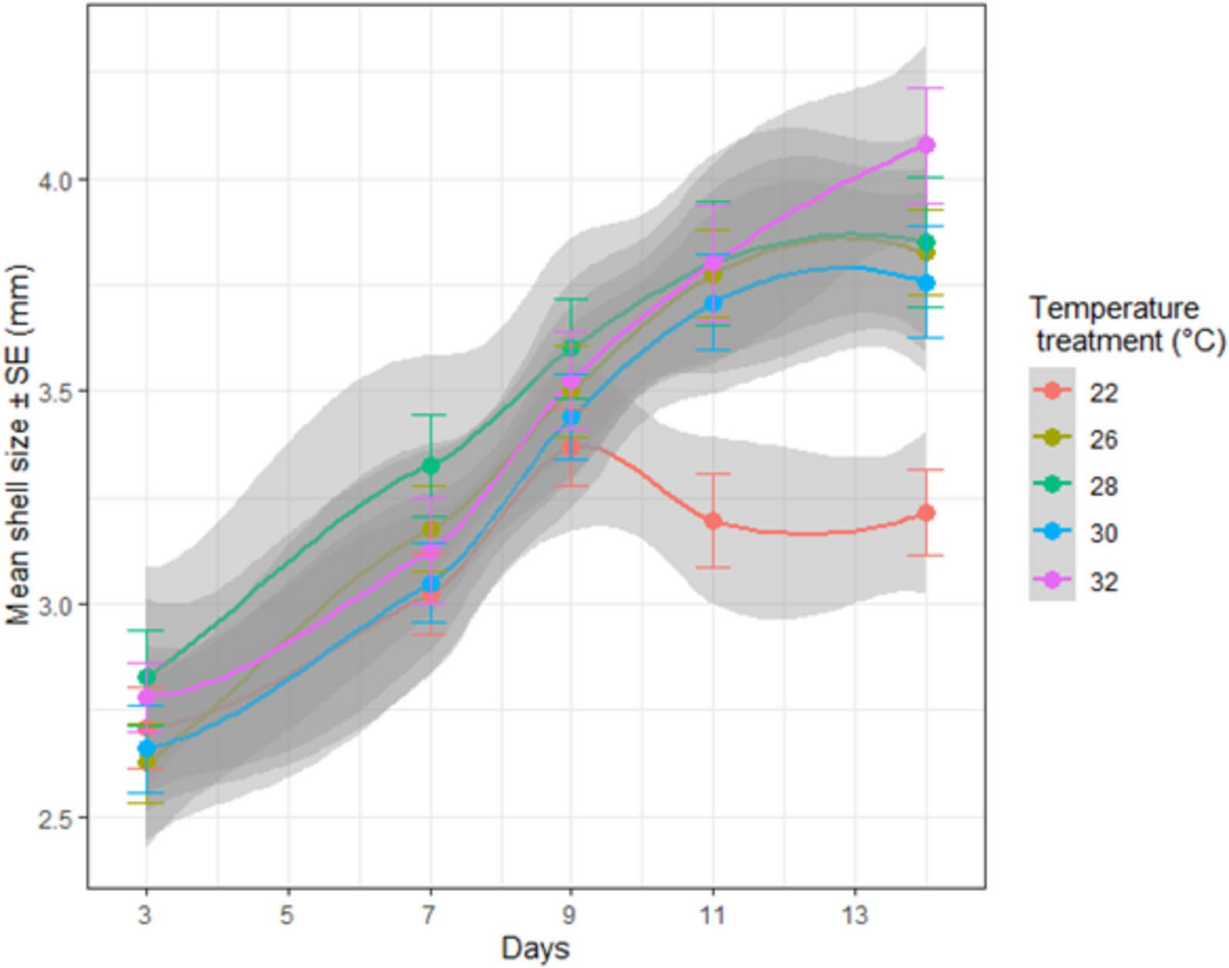
Shell size (mm) (mean ± SE) of juvenile Pacific oysters,* Crassostrea gigas*, at five temperatures from 22 to 32 °C across a 14 day period.

**Fig. 8:**
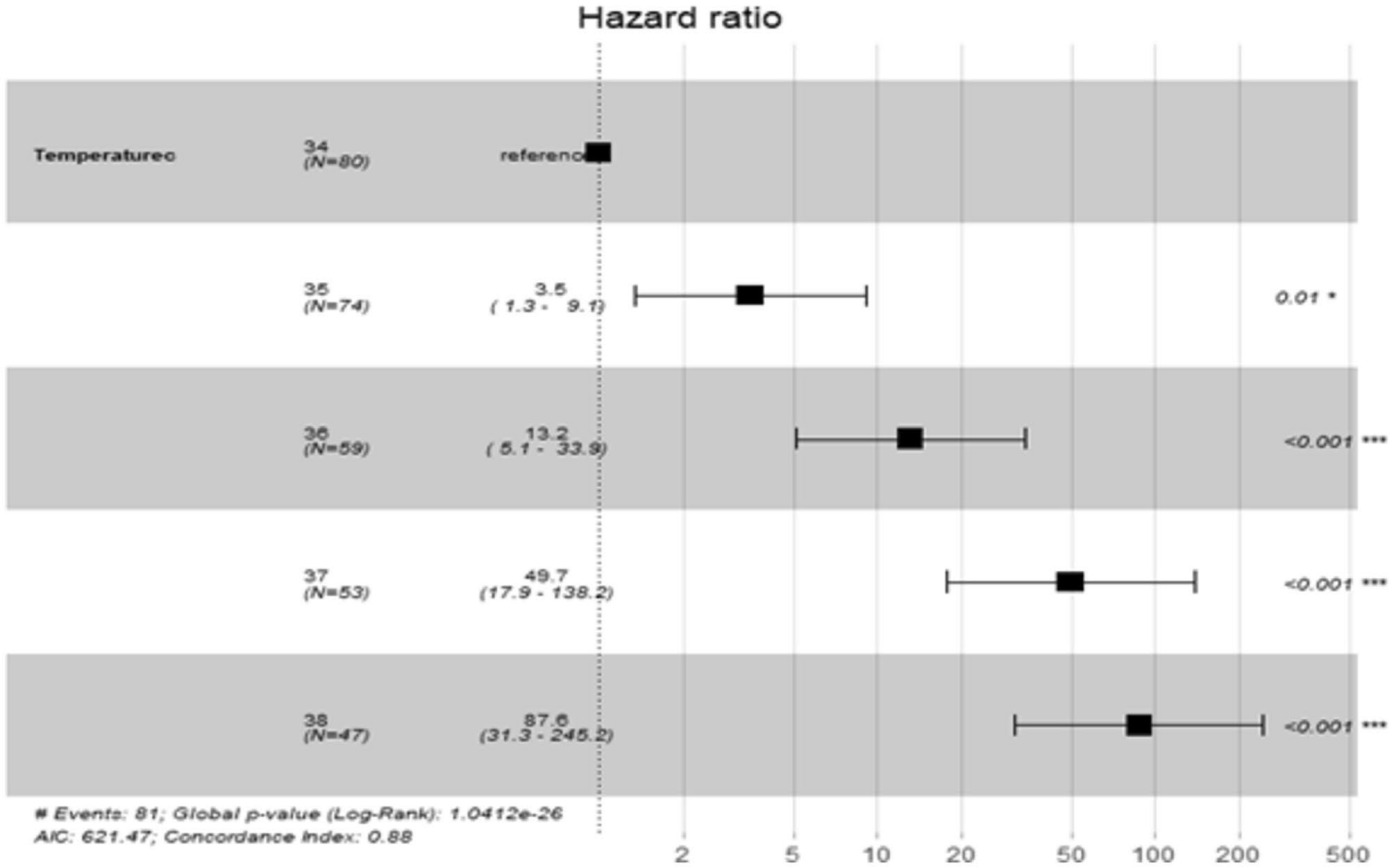
Forrest plot of hazard ± 95% Confidence Intervals, measured as mortality of juvenile *C. gigas* at 34 °C to 38 °C across the 5 day experiment (10/10/2023–14/10/2023). *P* values indicate significant differences compared to the 34 °C treatment. Only temperatures where mortality occurred were included.

In the moderate MWH survival of Pacific oyster juveniles was 100% for temperatures 22–28 °C for 14 days with only one mortality at 32° on day seven (Fig. 6, Table 1). While survival did not significantly differ among temperature, Pacific oyster juveniles were significantly smaller at the control temperature of 22° from day 11 onwards (Fig. 7, Table 4). In the extreme MHW there was 100% mortality of juvenile of *C. gigas* at temperatures of 37 °C and 38 °C by day three (Fig. 4). There was no mortality of juvenile of *C. gigas* at 34 °C and below (Fig. 4). Using Cox’s Proportional Hazard model, the risk of Pacific oyster juvenile mortality was significantly increased at 35–38 °C compared to 34 °C (Fig. 5, Table 5). The risk of mortality was 87.6 times greater at 38 °C compared to 34 °C (Fig. 8). Pacific oyster juveniles were significantly smaller in shell size at the higher temperatures between 35 and 38 °C than between 31 and 30 °C, juveniles were largest at 29 °C (Table 6). The LT_50_ value for Pacific oysters was 35.41 ± 0.14 at day 3, and 34.54 ± 0.14 at day 5.

**Table 4:** Shell size of juvenile Pacific oysters, *Crassostrea glomerata*, in a 14 day moderate MHW.

Day	7			11			14		
	MS	F	*P*	MS	F	P	MS	F	P
Temperature	0.67	1.72	0.14	2.71	5.02	0.000715***	3.55	6.70	4.49e−05***
Residual	0.39			0.54		0.54	0.53		
Post hoc	ns			30 = 32 = 30 = 28 = 26 < 22			30 = 32 = 30 = 28 = 26 < 22		

This was a single factor analysis of variance with temperature (29–38 °C) as a fixed factor. Significance labelled as *P* < 0.001 ‘***’, *P* < 0.01 **, *P* < 0.05*. Non significant differences are labelled as ‘ns’.

**Table 5:** Cox’s proportional hazard model measuring hazard as mortality of juvenile Pacific oysters, *C. gigas*, in an extreme MHW using 34 °C as the basis for comparisons with other temperatures.

Temperature °C	Coefficient	Hazard ratio	Std. Err. coefficient	z	P (z)	
35 °C	1.25	3.51	0.49	2.57	0.0102	*
36 °C	2.58	13.16	0.48	5.33	9.74e−08	***
37 °C	3.91	49.75	0.52	7.49	6.71e−14	***
38 °C	4.47	87.62	0.52	8.52	< 2e−16	***

Significance labelled as *P* < 0.001 ‘***’, *P* < 0.01 **, *P* < 0.05*. Non significant differences are labelled as ‘ns’.

**Table 6:** Analysis of Variances of shell size of juvenile Pacific oysters, *Crassostrea gigas*, with post hoc test in an extreme MHW.

	Df	SS	Mean Sq	F value	Pr(> F)
Temperature	9	13.42	1.49	3.95	7.59e−05***
Residuals	420	158.55	0.38		
Post hoc		38 = 37 = 36 = 35 = 34 = 33 = 32 < 30 = 31 < 29			

This was a single factor analysis using all time points with temperature (29–38 °C) as a fixed factor. Significance labelled as *P* < 0.001 ‘***’, *P* < 0.01 **, *P* < 0.05*. Non significances are labelled as ‘ns’.

### Thermal performance between species

**Fig. 9:**
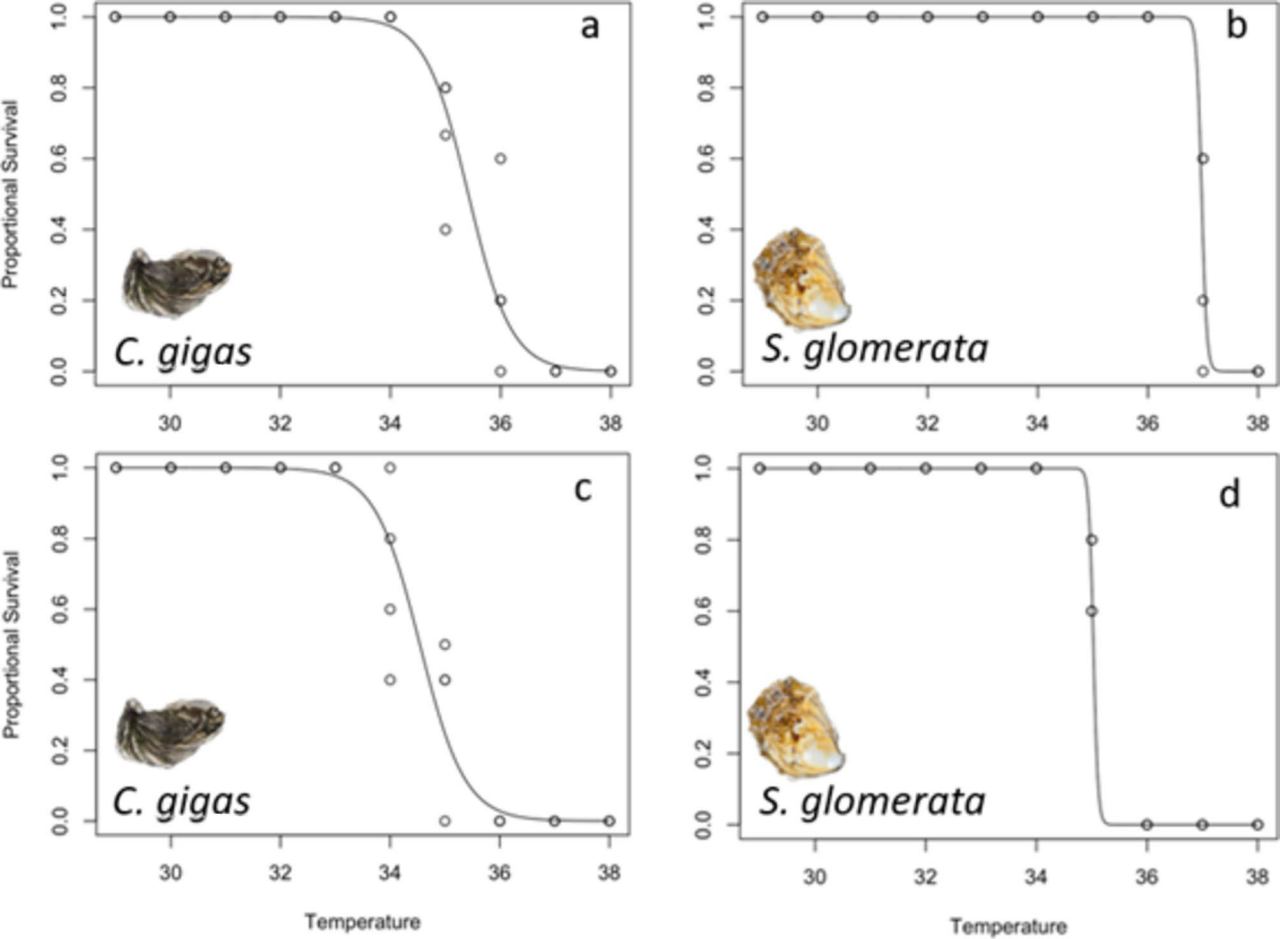
Thermal Performance Curves generated using a Generalised Linear Model (GLM) with a binomial distribution using survival data at 3 days (panel **a** and **b**), and 5 days (panel **c** and **d**) in the extreme MHW.

Thermal performance curves displayed a steep decline in Sydney rock oyster survival once at temperatures ≥ 35 °C (Fig. 9). Thermal performance curves showed a decline in survival of Pacific oysters around 35 °C. The modelled decline in survival was found to have been significantly less steep for Pacific, compared to Sydney rock oysters (Fig. 9).

## Discussion

Overall, we found an unequal impact of OW and moderate to extreme MHWs on the survival and growth of native Sydney rock oyster, *S. glomerata*, juveniles compared to the invasive Pacific oyster, *C. gigas*. In the OW and moderate MHW, temperatures of 32 °C significantly reduced the growth and size of juvenile Sydney rock oysters, while the survival and size of juvenile Pacific oysters were not affected at 32 °C. In contrast, growth of Pacific oysters was reduced at the lower temperature of 22 °C. While both oyster species experienced mortality in the extreme MHW at warming up to 38 °C, the greater LT_50_ values (day 3; 36.9 °C) of Sydney rock oysters demonstrates their capacity to survive temperatures of up to 35 °C, whereas 35 °C was largely lethal for Pacific oysters (LT_50_ day 3; 35.4 °C). Our findings suggest that juvenile Sydney rock oysters may be more resilient to extreme MHWs, however, Pacific oyster appear to maintain greater growth when faced with more OW and moderate MHWs.

These findings inform predictions on the future coexistence of *S. glomerata* and Pacific oyster. It is already suggested that Pacific oysters are competitively superior to Sydney rock oysters, this may continue to be the case in the temperate regions that Pacific oysters already occupy (Fig. 1). However, at the northern limits of distribution, Sydney rock oysters may retain an advantage due to their physiological capacity to survive and grow at temperatures up to and above 34 °C while at the critical and vulnerable juvenile stage. As estuaries along the south-eastern Australian coastline continue to warm^69^ Pacific oysters may still increase their intertidal distribution or latitudinal range and out compete the Sydney rock oyster. Pacific oysters are an invasive species that have already colonised habitats worldwide^56^and are expected to continue to increase their range across most of the Northwest European shelf, because of their capacity to tolerate warming seawater caused by climate change^72,73^.

In addition to the ongoing threat of MHWs, the Sydney rock oyster faces challenges within their estuary environments, including poor water quality^74,75^, increased disease and parasites^45,65^, and other impacts of climate change^76^. This study demonstrates that extreme MHWs will further challenge this ecologically and aquaculturally significant species. While it is known that adult oysters are better able to tolerate the more extreme experimental temperatures than juveniles, juveniles are also hardier than larvae, which are likely to be the more vulnerable life stage^68,77^. Outcomes for this species will likely depend on which life stage experiences the heatwave.

There are a number of explanations for the decreased growth of juvenile Sydney rock oysters at 32 °C compared to other lower temperatures. Temperature in general is a “master factor”^78^, influencing the physiology and behaviour of marine organisms. Thermal tolerance limits can be expressed as LT_50_ or the temperature at which 50% mortality occurred^79^. This thermal tolerance is strongly correlated with the maximal temperatures of their habitat with intertidal species being less able to increase their thermal tolerance^79^. We found the two species to have similar LT_50_ values, around 35 °C. Thermal performance curves, however, showed that Pacific oysters had much less abrupt decline in survival compared to Sydney rock oysters, perhaps indicating the potential for adaptation to elevated temperatures. While their decline at extreme temperatures was abrupt, Sydney rock oysters appear to be able to survive up to 36 °C, where Pacific oysters could not.

Where both species are present, Sydney rock oysters occupy higher intertidal habitats than Pacific oysters^80^. Inhabiting the higher intertidal zone comes with physiological costs which are associated with their need to maintain a thermal tolerance limit higher than subtidal counterparts^79,81^. For example, one such physiological process that limits thermal tolerance is the limited capacity of both ventilation and circulation processes when faced with extreme temperatures is known at as the “oxygen-and-capacity-limited thermal tolerance” (OCLTT) hypothesis^82^. This hypothesis explains that elevated seawater temperatures will impact ventilation and circulation processes in marine organisms which will combine with decreased oxygen in water at higher temperatures and increased metabolic rates^83^. While this hypothesis has received some attention, ultimately, the ability of oysters and other marine ectotherms to survive MHWs will depend on their physiological plasticity and ability to adapt to a more thermally variable habitat^84^.

The optimal temperature for Sydney rock oyster juveniles in culture was identified to be 30 °C^66^ and so the higher temperatures used here was likely to be above their critical thermal maximum thereby causing decreased growth at 32 °C. Studies on Sydney rock oysters exposed to atmospheric heatwaves of 50 °C found variation in survival between 25 and 60% depending on genetically distinct family lines^69^ Both the Sydney rock and Pacific oyster have a considerable proportion of environmental stress genes in their genomes^85,86^ although how this translates to stress tolerance is not known. Sydney rock oysters have demonstrated significant phenotypic plasticity in coping with a variable environment. For example, Sydney rock oysters were able to adjust metabolic rates when moved from the subtidal to intertidal zones^81^. Similarly, Pacific oysters have also demonstrated a similar capacity for phenotypic plasticity in the intertidal zone^49^. This degree of plasticity is suspected to originate because the offspring of broadcasting spawning sessile organisms can settle and metamorphose in a diversity of environments, including those mis-matched to their parents^87,88^.

We found Pacific oysters to grow faster at MHW temperatures, however, whether this leads to Pacific oysters outcompeting Sydney rock oysters is still open to debate. Resilience to temperature alone will not determine this outcome. Other factors such as exposure to atmospheric heat may also determine responses. Sydney rock oysters are believed to be “hardier” when exposed to increased air temperature compared to Pacific oysters, being able to shut their valves for up to two weeks^77,80,89^. Previous research on the tolerance of adult Sydney rock oysters to aerial exposure has demonstrated they can tolerate atmospheric heat waves of up to 50 °C over two hours^69,90^. Such tolerances to air exposure are correlated with current differences in distribution between Sydney rock and Pacific oysters with *C. gigas* being mainly a low intertidal to sub tidal species^80,91^. Along the south-eastern mainland Australian coastline, Sydney rock oysters are more commonly found in the intertidal and in areas where both Sydney rock and Pacific oysters occur, Pacific oysters remain a low percentage of the total abundance^46^. Studies investigating the impact of aerial exposure on oysters have found that both increased temperature and duration of aerial exposure reduced survival and growth of *Ostrea angasi* and acted as limits to the distribution in the intertidal and restoration^92^. Similarly, Heo et al.^93^ found that aerial exposure of intertidal Pacific oysters to 45 °C for 4 h a day affected their survival with total mortality of individuals after 6 days, and concluded that high temperatures during emersion may cause mass mortality and disease in oysters.

In addition to aerial exposure, disease is likely to play a role in oyster survival following a MHW^90^. In a study contradicting our findings, it was shown that juvenile Pacific oysters (6 mm shell length) experienced 77% mortality at 26 °C^29^. In Green et al.^29^ the mortality was determined to be triggered by bacterial disease, likely caused by bacteria from the *Vibrio* genus. Sydney rock oysters, however, can acclimate to heatwave conditions and reduce mortality caused by *Vibrio* bacteria^90^. Further experiments are needed to determine species differences in responses when MHW are combined with other environmental stressors including aerial exposure and disease. Thought needs to be taken in experimental design to mimic the real variable world of an oyster as much of our current understanding on the thermal range of both species is based on experiments with oysters fully submerged and optimally fed.

This study fills a current gap in knowledge about how OW and MHWs will affect the juvenile stages of the main ecologically and economically significant oyster species in Australia. Our results suggest that Pacific oysters have more resilience than Sydney rock oysters when faced with moderate MHWs, but Sydney rock oysters can hang-on at extreme temperatures. However, these results are based solely on the effect of MHWs and temperatures of 24–38 °C rather than the real multiple stressor world of an estuary where oysters experience fluctuations such as emersion in air, acidification (pH), salinity, nutrients and disease. As MHW events and precipitation become more frequent due to a changing climate, it is important to understand how oysters and other marine organisms will respond to these events. As their estuarine battlegrounds warm, and MHWs become more frequent, the greater thermal tolerance of Sydney rock oysters or the greater growth of Pacific oysters may determine how these versatile organisms are distributed. As changes in oyster distribution will alter the function, and the types of food that can be grown in Australian estuaries the potential survival and range change of the two species is crucial to understand. Finally, climate change is going to change the way that invasive and native species interact. It is therefore imperative that we understand how MHWs both moderate and extreme will alter interactions which affect estuarine and marine foundation species and ecosystem engineers and those vitally important to aquaculture.

## Methods

### Organism and maintenance

The Sydney rock oyster has its warm range edge in Harvey Bay, Queensland and its cold range edge around the NSW/Victoria border^89^ (Fig. 1). For Pacific oysters the northern range edge is approximately the Hastings River and cool range is southern Tasmania (Fig. 1)^89,94^.

A moderate and extreme MHW were simulated to measure the survival and growth of juvenile (spat) of Sydney rock and Pacific oysters. Approximately 400 *S. glomerata* juveniles (1200–2000 μm shell length) were sourced from Camden Haven Oyster Suppliers Pty Ltd (31.6502° S, 152.7967° E) and approximately 400 Pacific oyster, *C. gigas*, juveniles (1200 μm shell length) were sourced from East Coast Oyster Nursery Pty Ltd (− 35.701133° S, 150.171915° E). The rearing temperature history of both species was 20–22 °C. The oysters were acclimated in the lab for 1–2 days in 1 μm filtered seawater (FSW) in a 4L beaker at 22 °C. During all acclimation and experimental exposures (see below), oysters were fed live algae cultured comprising of 50% *Chaetoceros calcitrans and 50% Isochrysis galbana* at a rate of 1 × 10^6^ cells oyster^−1^ day^−1^.

### OW and MHW

To investigate the responses of Sydney rock and Pacific oysters to OW and MHW following Hobday et al.^6^ and Hobday et al.^95^ and Ewere et al.^96^ we exposed Sydney rock and Pacific oysters to a moderate MHWs defined as 2–4 °C degrees above the thermal range that lasted longer than 5 days. This also followed Ewere et al.^96^ who exposed Sydney rock oysters to a MHW of 5 °C above ambient sea surface temperature where maximum SST of 21–22 °C which are typical temperatures of cultivation locations of Sydney rock^97^. For Pacific oysters the median temperature of where they are found in Australia is between 20 and 25 °C^94,98,99^.

Two scenarios were used (1) OW and a moderate MHW as described by Hobday et al.^95^ as category (1) and (2) an extreme MHW, category IV^95^. For the OW, moderate MHW the oysters were exposed to a temperature range of 22–32 °C for two-weeks. For the extreme MHW the range of temperature was between 29 and 38 °C for one week (Table 1). These simulated MHWs emulated recent OW and MHW in the region and beyond and allowed us to determine the upper thermal tolerance range which has been estimated to be 30 °C for the Sydney rock oyster^96^ and 32 °C for Pacific oysters^98^.

The experimental temperature range of the OW and moderate MHW was between 24 and 32 °C for the Sydney rock oyster and between 22 and 32 °C for the Pacific oysters, given their lower thermal distribution range (Fig. 1). The lower temperatures were used as the control temperatures. The experimental temperature range of the extreme MHW was between 29 and 38 °C at the upper end of both species thermal range (Table 1).

Temperature treatments for the heatwave experiment were created using two aluminium blocks set up in parallel that allowed for a stable, static thermal gradient established by using warm and cold-water inputs at either end. Each heat block had four columns of holes (20 mm diameter) to fit the vials (40 mL), with each row representing a designated temperature treatment.

The set up for the simulated OW and moderate MHW was as follows. For Sydney rock oyster juveniles, there were four temperatures at 2–3 °C intervals between 24 and 32 °C (i.e. 24 °C, 26 °C, 29 °C, 32 °C) in the temperature block with eight replicate vials at each temperature, and five juvenile spat within each vial, for a total of 160 juveniles. For Pacific oyster juvenile spat, there were five temperatures at 2–3 °C intervals between 22 and 32 °C (i.e. 22 °C, 24 °C, 26 °C, 29 °C, 32 °C), with eight replicate vials, five juvenile spat within each vial, for a total of 200 juveniles (Table 1). Oyster juvenile spat were selected at random from the source container, checked if they were alive and placed in the vials. To feed the oysters 1 ml of a 50/50 algal combination of *Chaetoceros calcitrans* and *Isochrysis galbana*, (~ 1,200,000–1,500,000 algal cells) was placed in the vials daily and the water was renewed with water at temperature every second day commencing at day 3. On these days the oysters were removed from the vial and placed in a well plate to check survival, measured using a graticule eyepiece under a dissecting microscope every second day and promptly (within 2–3 min) placed into a new vial containing FSW at experimental temperature and returned to the temperature block. Water temperature and dissolved oxygen (DO) levels were measured daily using a WTW Multi3420 salinity and temperature probe, and a Eutech DO 6 + Portable Dissolved Oxygen Meter using a 1-point calibration mode (mg/L) (Supplementary Table 1).

The same method was followed for the extreme MHW but with a higher temperature range (29–38 °C). For Sydney rock and Pacific oysters, there were 10 temperature intervals from 29 to 38 °C with intervals of 1 °C (Table 1). Each temperature had four replicate vials with five oyster juveniles in each, giving a total of 200 oysters (Table 1). While upper temperatures were high, they were within the tolerance limits of oysters previously tested for short periods of aerial exposure^68^. Experiment duration was approximately a week, or six and five days for the Sydney rock and Pacific oyster respectively.

### Statistical analyses

Data on shell size for Sydney rock and Pacific oysters were analysed with a single factor Analysis of Variance (ANOVA) with temperature as a fixed factor. Data were also checked and confirmed to meet heterogeneity of variances and normal distributions, critical assumptions of ANOVA^100^. Cox’s proportional hazard regression model was also used to determine the risk of the hazard of mortality across temperature ranges and days, until day 5 using the “Survminer” package. A Forrest plot was created to visually represent the hazard ratio. All LT_50_ values were determined for each oyster species by calculating a Generalised Linear Model (GLM) with a binomial distribution using survival data from days 3 and 5 in the extreme MHW (MASS package^101^) data analyses were done using RStudio 4.2.3^102^.

## Supplementary Information

Supplementary Table 1.

## Data Availability

The datasets used and or analysed in this study are available from the corresponding author on reasonable request.
